# An Evaluation of the Outcomes of Mutual Health Organizations in Benin

**DOI:** 10.1371/journal.pone.0047136

**Published:** 2012-10-15

**Authors:** Slim Haddad, Valery Ridde, Ismaelou Yacoubou, Geneviève Mák, Michel Gbetié

**Affiliations:** 1 Research Centre of the University of Montreal Hospital & Department of Social and Preventive Medicine, Faculty of Medicine, University of Montreal, Quebec, Canada; 2 Centre d’études et d’appui technique aux institutions de micro assurance santé, Parakou, Benin; 3 Centre National Hospitalier et Universitaire de Cotonou, Cotonou, Benin; Groningen Research Institute of Pharmacy, United States of America

## Abstract

**Background:**

Mutual health organizations (MHO) have been seen as a promising alternative to the fee-based funding model but scientific foundations to support their generalization are still limited. Very little is known about the extent of the impact of MHOs on health-seeking behaviours, quality and costs.

**Methodology/Principal Findings:**

We present the results of an evaluation of the effects attributable to membership in an MHO in a rural region of Benin. Two prospective studies of users (parturients and hospitalized patients) were conducted on the territory of an inter-mutual consisting of 10 MHOs and as many healthcare centres (one, Ouessé, serving as a referral hospital) and one hospital (Papané). Members and non-members were matched (142 pairs of parturients and 109 triads of hospitalized patients) and multilevel multiple regression was used. Results show that member parturients went to healthcare centres sooner (p = 0.049) and were discharged more quickly after delivery (p = 0.001) than non-members. Length of stay in some cases was longer for hospitalized member parturients (+41%). Being a member did not shorten hospital stay, total length of episode of care, or time between appearance of symptoms and recourse to care. Regarding expenses, member parturients paid one-third less than non-members for a delivery. For hospitalized patients, the average savings for members was around $35 US. Total expenses incurred by patients hospitalized at Papané Hospital were higher than at Ouessé but the two hospitals’ relative advantages were comparable at −36% and −39%, respectively.

**Conclusion/Significance:**

These results confirm mutual health organizations’ capacity to protect households financially, even if benefits for the poor have not been clearly determined. The search for scientific evidence should continue, to understand their impacts with regard to services obtained by their members.

## Introduction

Community-based health insurance, known in francophone Africa as mutual health organizations (MHO), has been supported by the convergent commitments of governments and development agencies, and is seen as a promising alternative to the fee-based funding model inherited from the Bamako Initiative. In its 2010 report on health systems financing, WHO reinforced this position by emphasizing the role of community-based insurance mechanisms as a strategy that could complement other existing forms of social insurance to achieve universal coverage [1].

Expectations regarding community-based insurance remain high. However, scientific foundations to support its generalization are still relatively limited. Systematic reviews have not yet managed to provide solid evidence about their benefits, partly because of the limited number of robust studies published to date in the scientific literature [2]. Evaluations often tend to be based on observational designs of limited internal validity [2], [3], [4], and to describe experiences that were successful or that relate to surviving organizations that may not be representative [3].

Nevertheless, even with these limitations, systematic reviews tend to report the following patterns: belonging to an MHO: (1) increases utilization of health services during episodes of illness [5], [6], [7], [8], [9], [10], [11], [12], [13], [14], [15], [16], [17], [18], [19], [20], [21], [22]; (2) reduces costs for an episode of care [5], [7], [9], [14], [20], [23] and; (3) lessens exposure to catastrophic expenses [5], [24]. Yet the evidence is inconsistent and the size of the observed effects is sometimes modest. We still know little about the potential advantages of MHO membership with respect to duration of episodes of care, distances travelled by patients, delays before seeking care, and user satisfaction.

Little is known as yet about the extent of the impact of community-based insurance on health outcomes, nor on access and quality of health care services. In this article we present the results of an evaluation of the benefits attributable to membership in a mutual health organization in a rural region of Benin. As in other countries of the region, the MHO movement in Benin is gaining significant ground. There are at least seven active MHO networks, encompassing a total of about 135 MHOs, and the authorities are considering the possibility of including them in a national health insurance system [25].

## Methods

### The Intervention and the Evaluability Analysis

The intervention was carried out mainly in a rural zone in the country’s central and northern areas. The network had about 30 MHOs. The model was based on a local cooperative model in which villagers joined together in mutualist groups that, in turn, collectively formed village MHOs. New MHOs were integrated into the existing MHO network, a strategy that helped achieve economies of scale and increase the insurance pool, to reduce each organization’s financial risks. The membership fee was paid to the MHO annually and was, at the time of the study, between 1500 and 2000 F CFA per person ($1 US≈500 F CFA). The risks covered varied somewhat from one MHO to another. All of them covered deliveries, minor surgery, and hospitalization. Women could deliver in a primary care centre or go directly to a maternity referral facility. All MHOs in a hospital’s catchment area were combined into one “inter-mutual”. This entity carried out some of the management tasks and the interface with referral hospitals: resolving conflicts, sorting out disputes, negotiating contracts, etc. Each MHO signed a service agreement with a healthcare centre, generally located within the village itself. The MHO covered a predetermined portion of the cost of the episode of care, as long as this was provided by one of the healthcare centres or one of the two hospitals under contract. Taking into account deductibles and the set limits for payment, the MHOs’ share represented 60% to 75% of the total cost billed to users by healthcare facilities. The MHO paid the healthcare centre directly for its share (cashless system), and patients paid their share on discharge.

The study was conducted on the territory of an inter-mutual consisting of 10 MHOs and as many healthcare centres and one hospital. One of the centres (Ouessé) also served as a referral hospital, with a unit for hospital admissions and a maternity unit. The hospital in Papané was a charity organization offering a wide range of services in medicine, pediatrics, surgery, and gynecology-obstetrics. The mean distance between the hospital and the villages was 36 km (maximum 59 km). At the time of the study, about 8% of the population in this zone belonged to an MHO; each MHO had around 700 members, on average.

An exploratory analysis of the impacts of these MHOs on community dynamics and members’ empowerment has already been published [26]. Here we present the impacts observed on MHO members who used services. The evaluation was conducted by the team’s investigators (SH, VR, MG), who were independent of the intervention, were not involved in the management or development of the MHO network, and had no incentive to report positive results. The evaluation was preceded by an evaluability analysis, including consultation with local actors, documentary analysis, and field visits to reconstruct the logic model of the intervention [27] and prepare the study design. Outcome indicators were chosen based on the intervention’s core hypothesis, which was that membership in the MHO would reduce members’ vulnerability by lessening the cost constraints encountered throughout the episode of care, including: (1) access difficulties and delays in health-seeking behaviours before contacting the healthcare facility; (2) reception, length of stay, and services provided during the hospital stay; and (3) expenses incurred at the end of the episode of care.

### Study Design

The study design was based on two prospective studies of users–parturients and hospitalized patients–some of whom were members of MHOs, and others, not. The study included all the facilities in the area (n = 11) contracted out by the MHO network. To strengthen the comparability of the groups and reduce self-selection biases, members and non-members were matched according to some key characteristics. Given the funding available for this study, the observation period was limited to 12 months. The feasibility study established a one-year recruitment scenario of 135 parturients and 100 hospitalizations among the members. The outcomes indicators are presented in Table 1.

**Table 1:** Outcomes indicators.

*Delays and lengths of stay*	*Difficulties encountered in accessing care*	*Assessment of care received at the centre/hospital*	*Expenses*
Time spent before going to the centre or hospital	Did not have the moneyrequired to pay for care	Received good care	Expenses prior to going to the centre or hospital
Length of stay at the centre and/or hospital	Was prevented from going to the hospital*	Was well received	Medical expenses billed at the cashier's desk of the centre or hospital
Delay before transfer to hospital*	Had to postpone seeking services	Was happy with the care received "overall"	Additional medications purchased
	Was unable to buy certain prescribed medications	Would be willing to go back	Transportation expenses
	Had to postpone buying certain medications		Food expenses
			Total cost of care at the centre/hospital**
Total duration of episode of care**	At least one of the preceding situations**	Assessment index for care received**	Total cost for the episode of care**

* Questions relevant only for persons referred from a healthcare centre to a hospital.

** Indicators calculated from the responses to the preceding questions.

The study was approved by the ethics committees of the University of Parakou (Benin) and of the Research Centre of the University of Montreal Hospital Centre (Canada). It was presented to and approved by the Regional Office of the Ministry of Health. Written informed consent from all participants was obtained.

### Data Collection and Analysis

Data was collected at two points in time. The first data collection took place in the healthcare facilities. All MHO members admitted during the observation period were identified from the admissions registries. Information on the episode of care (key dates, transfers, costs, diagnoses, etc.) were transcribed onto an observation sheet. We then matched these member patients with non-members. In the case of parturients, the criteria for matching were: the healthcare facility used, the type of delivery (normal, dystocic), the women’s provenance (village or area of residence) and the date (or the date of the closest delivery in time occuring in the health facility). For hospitalized patients, the criteria were: circumstances of admission (direct or referral from a healthcare centre), site of hospitalization (Papané or Ouessé), unit (medicine, surgery, pediatrics, gynecology), age group, and gender. One-to-one matching was done for parturients, and one-to-two for hospitalized patients. Due to large variations in hospital case mix, we expected greater heterogeneity within the hospitalized group. Therefore, to increase the power and improve statistical efficiency, we used two controls per case [28], [29]. To reduce the risks of interference in data collection, the subjects recruited were not met during their stays, and the care teams were not involved in the selections from the registries. In a second phase, surveyors located the patients and visited them in their homes (between three days and one month post-discharge). Their consent (and that of the person in charge of the household) was sought. Consenting persons were questioned about the circumstances of the episode of care. In the case of a child, the mother or the best-informed person responded. The recruitment led to the selection of 142 pairs of parturients and 109 triads of hospitalized patients.

Two-sided statistical tests for matched observations were used to explore outcome differences between members and non-members. The net gain or change, rather than the benefit associated with the condition of membership, was estimated for each outcome indicator using multilevel multiple regression, taking into account the nested structure of the observations (patients nested in pairs or triads that were nested within health facilities). All analyses were conducted with Stata [30]. Covariables were introduced into the models to further correct for potential differences in healthcare centre case mixes and to minimize MHO membership self-selection biases. They were: (i) occupation, and distance between home and hospital (km), for hospitalized patients; and (ii) occupation, parity, age, and occurrence of obstetrical complications, for parturients. Cost variables were modelled with and without logarithmic transformation because of their asymmetric distribution. Assessment of access-related benefits was based on an aggregate indicator: occurrence of at least one of the difficulties mentioned in the interview questionnaire (Table 1). The assessment of care received was based on an aggregate score obtained by correspondence factor analysis (responses to the relevant questions were formulated using a 5-point Likert scale).

The intervention’s impact was assessed using the value of the marginal effect associated with the condition of membership after matching and statistical adjustment for covariates. The models included terms of interaction in order to differentiate the marginal effects of the intervention according to whether the parturient: (i) used only one healthcare centre; (ii) was transferred from a healthcare centre to a referral maternity hospital; or (iii) went directly to one of these maternity units. A similar approach was used to estimate the effects of MHO membership according to whether patients were hospitalized in one of the two hospitals.

## Results

### Group Comparability

The parturient pairs consisted mostly of women who had used only a healthcare centre (67%); 25% were admitted directly to the maternity units of the Ouessé or Papané hospitals, and 8% were admitted to a centre and then transferred to one of these maternity units. Three-quarters of the deliveries were normal. With respect to the hospitalized patients, differences were observed in the triads for two matching criteria (hospital unit and patient gender). These were modest differences and not significant, but they were taken into account in the statistical modelling. Children under the age of five years made up more than half the sample (55%). The proportion of patients referred by a healthcare centre was 8%, and 50% of those were females. Table 2 shows the comparability of members to non-members with respect to other characteristics than those used for matching. The profile of member parturients was identical to that of non-members except for occupation. This variable was subsequently included in the list of modifying factors for multiple regressions. Among hospitalized patients, members and non-members were comparable for all criteria considered.

**Table 2:** Comparison of member and non-member groups.

Group		Members (%)	Non-members (%)	p-value
*Hospitalized patients*				
	Age			0.12
	0–5	52.3	56.0	
	6–15	11.9	5.5	
	16+	35.8	38.5	
	Sex			0.15
	Female	45.9	52.8	
	Reported occupation (16 years and older)			0.568
	Farmer	17.9	25.0	
	Housewife	17.9	21.4	
	Artisan	17.9	25.0	
	Shopkeeper	30.8	17.9	
	Other	15.5	10.7	
*Parturients*				
	Age			0.45
	16–20	20.4	23.9	
	21–35	73.9	67.6	
	≥36	5.6	8.5	
	Reported occupation			0.032
	Farmer	17.6	19.0	
	Housewife	23.2	31.0	
	Artisan	16.2	22.5	
	Shopkeeper	38.0	21.1	
	Other	5.0	6.4	
	Parity			0.8
	Nullipara	16.2	17.7	
	Primipara	28.2	24.8	
	Multipara	55.6	57.4	
	Person who followed the pregnancy			0.29
	No one	53.9	46.1	
	Family, friends	38.3	47.5	
	Health professional	2.8	1.4	
	Other	5.0	5.0	
	Occurrence of a complication at delivery			0.62
	No complication	85.9	83.8	

### Differences between Members and Non-members

#### Problems encountered in accessing care and services

**Figure 1:**
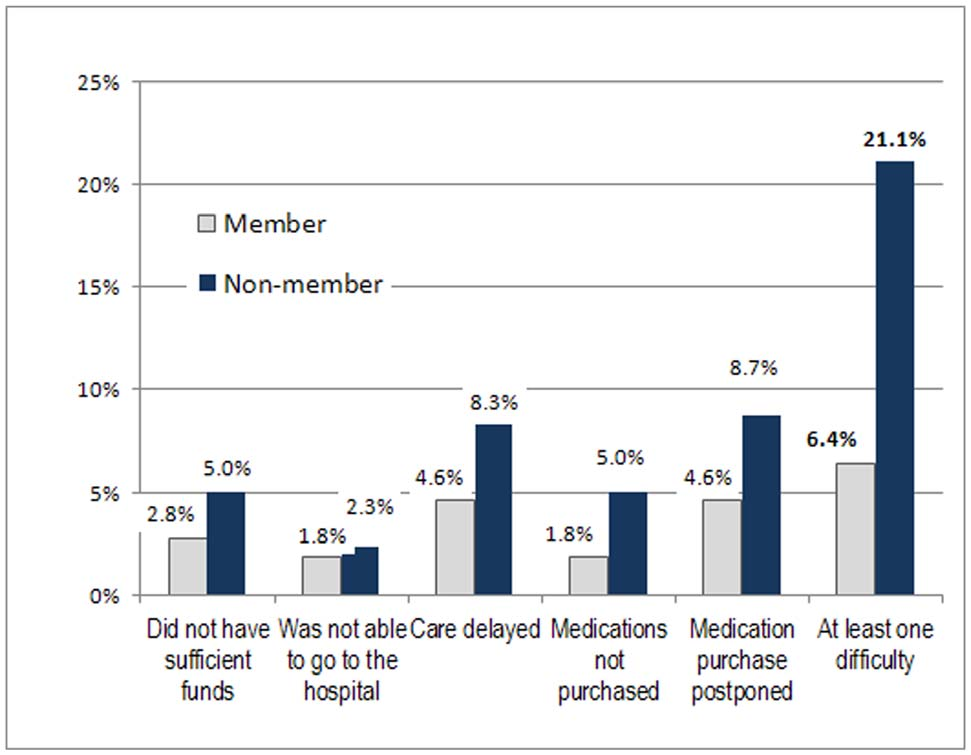
Difficulties encountered by hospitalized patients according to their status.

The proportion of hospitalized patients who reported experiencing difficulties in access was low in both groups (Figure 1). Members reported fewer problems, but differences with non-members were only significant after aggregation of responses, when comparing the proportion of respondents reporting having had at least one such problem (67% among members and 21% among non-members). None of the 141 member parturients appeared to have been affected by the difficulties mentioned in our questionnaire. Among non-members, fewer than 5% encountered problems.

#### Delays and lengths of stay

Member parturients went to the healthcare centre sooner (on average, nearly five hours sooner; p = 0.049) and were discharged more quickly after delivery (on average, nearly 10 hours sooner; p = 0.001). On the other hand, length of stay in one of the two referral maternity units was two days longer for hospitalized member parturients than for non-members (+41%; p = 0.006). For hospitalized patients, being a member did not shorten hospital stay, total length of episode of care, or time between appearance of symptoms and recourse to care (results non-significant, not presented).

#### Assessment of care and services received

The differences in the hospitalized patients’ assessments of care received were not significant (results not presented in order to limit the number of graphs and tables). Members and non-members had very favourable and very similar opinions, with only one exception: MHO members considered the reception at Ouessé to be less positive. The parturients’ level of satisfaction was high (above 85% for each indicator explored) for both members and non-members.

#### Expenses

**Figure 2:**
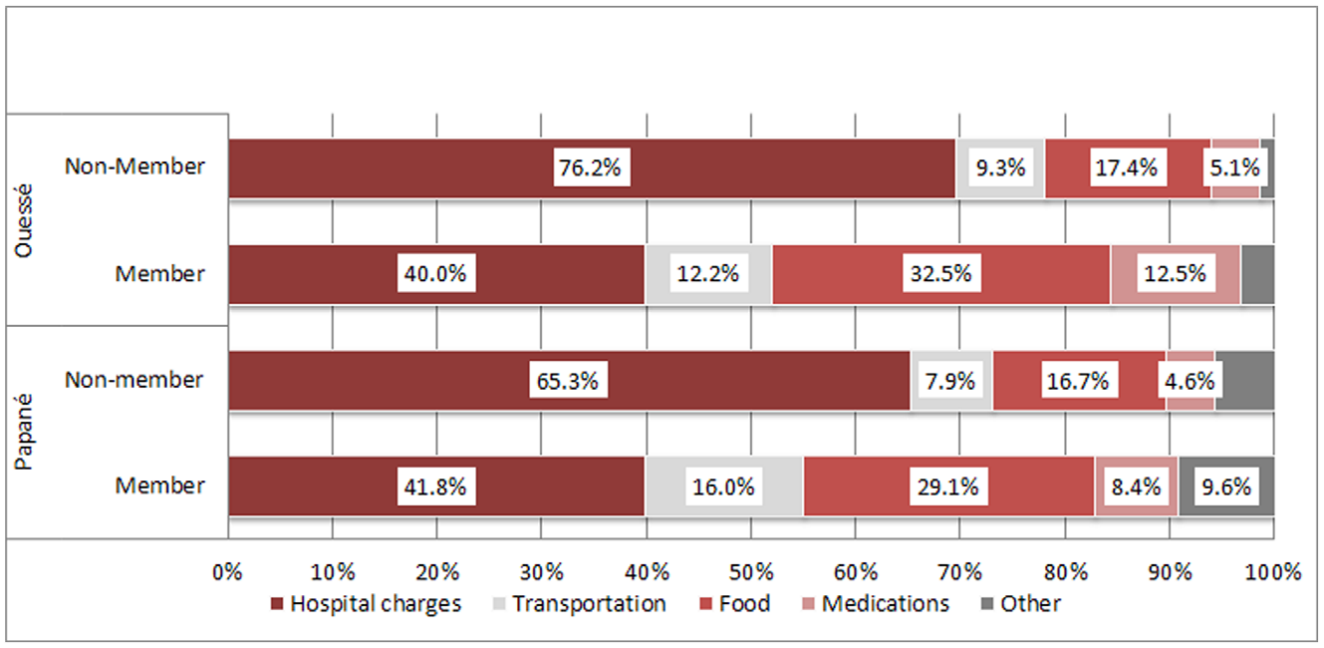
Distribution of hospitalization expenses by line item according to user status and healthcare facility.

Estimates include all that was spent during the episode of illness for the parturient or the patient hospitalized, whether the source of the funds was the person herself, her family or another source. The burden of expenses was, on average, significantly and substantially reduced among members (Table 3). Member parturients paid one-third less than non-members for a delivery, whether or not it was followed by hospitalization. Total expenses incurred by patients hospitalized at Papané Hospital were naturally higher than at Ouessé, which is only a referral centre, but the relative advantages were comparable at the two hospitals (−36% and −39%, respectively). The savings achieved by MHO members were naturally reflected in the distribution of hospital expenses by line item. For non-members, about two-thirds of the expense burden was in hospital charges. This proportion was considerably lower for members, since a portion of their hospital costs was covered by the MHO (Figure 2).

**Table 3:** Delays in health-seeking behaviours, length of stay, and expenses (F CFA) of members and non-members*.

*Parturients*	Episodes using only a healthcare centre			Episodes including a stay in a hospital maternity unit		
	Member	Non-member	Difference	Member	Non-member	Difference
Time before arrival at centre/maternity unit (days)	**0.47**	**0.67**	**−0.20**	0.68	0.74	
Length of stay at healthcare centre (days)§	**1.64**	**2.07**	**−0.43**	0.47	0.28	
Delay before transfer to maternityunits (days)§§				0.57	1.67	
Length of stay. hospital maternity unit (days)				**7.4**	**5.3**	**2.15**
Charges billed at healthcare centre (F CFA)	**8 682**	**12 918**	**−4 236**	3 960	3 130	
Charges billed at maternity unit (F CFA)				**32 605**	**49 579**	**−16 974**
Total expenses episode of care (F CFA)	**8 821**	**12 949**	**−4 128**	**33 549**	**50 407**	**−16 858**
***Hospitalized patients***	**Hospitalized at Papané**			**Hospitalized at Ouessé**		
	**Member**	**Non-Member**	**Difference**	**Member**	**Non-Member**	**Difference**
Charges billed at hospital (F CFA)	**8 997**	**25 718**	**−16 721**	**5 245**	**19 383**	**−14 138**
Total expenses, Hospital stay (F CFA)	**27 464**	**43 939**	**−16 475**	**16 148**	**27 317**	**−11 169**

§ Parturients who used only a healthcare centre.

§§ Parturients transferred from a centre to a hospital maternity unit.

* Paired tests. Only significant differences reported. Boldface: significant difference.

### Estimate of the Impact of MHO Membership

**Figure 3:**
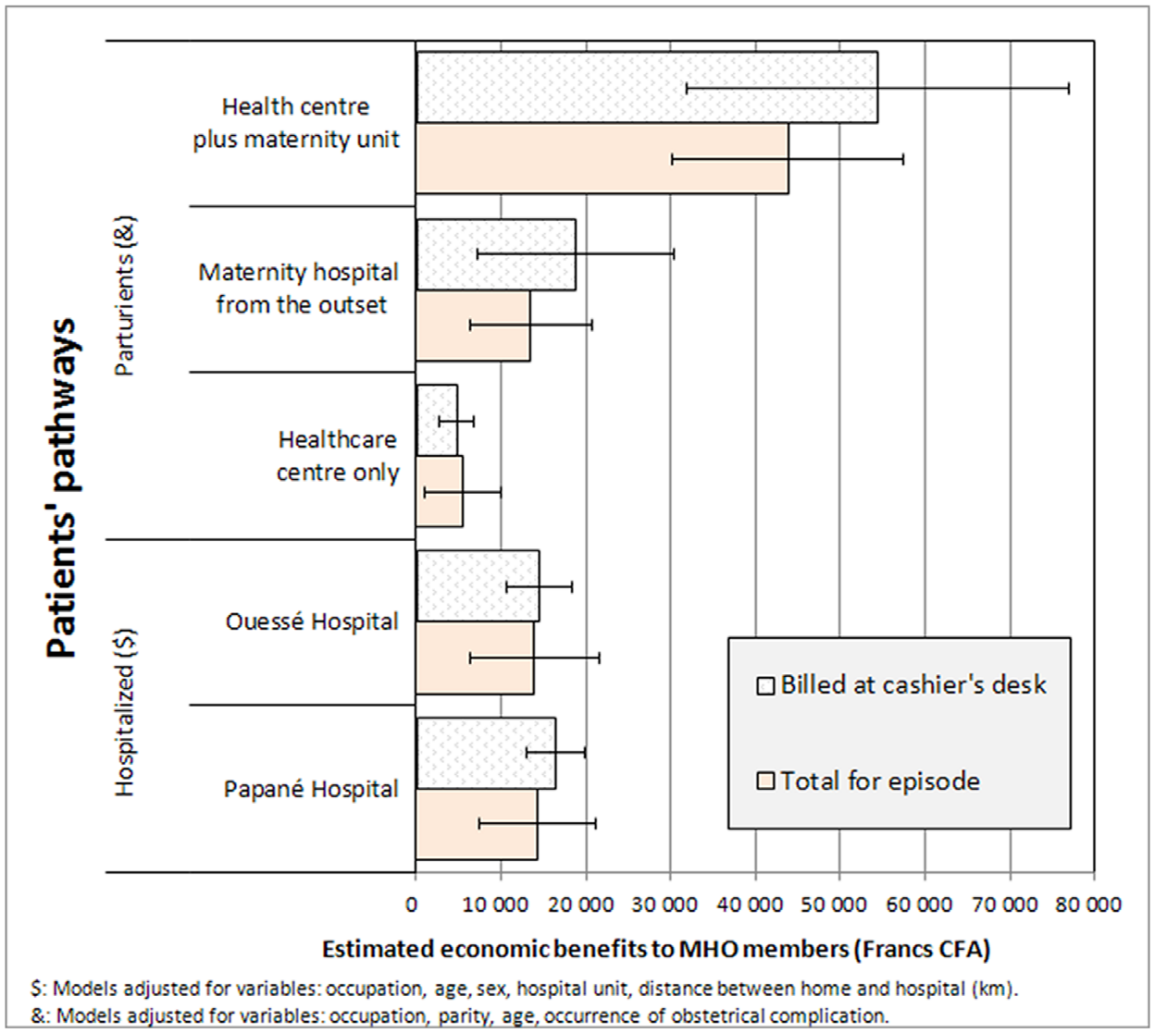
Economic benefits attributable to MHO membership according to patients’ care pathways (in Francs CFA).

There were, after adjustment, no differences between members and non-members with respect to difficulties in obtaining services or accessing care. There was also no advantage to either parturients or hospitalized patients in terms of accelerated health-seeking behaviours, length of stay in a health facility, or total duration of the episode of care. Figure 3 shows the estimate of the savings attributable to being an MHO member, after multivariate modelling, controlling for healthcare centre case mix distribution and socioeconomic conditions. The models suggest that in both cases, whether delivery or hospitalization, members experienced significant savings. For deliveries, savings varied substantially depending on the women’s care path. For the 8% of women seen first at a healthcare centre and then transferred to a better equipped maternity unit, the savings (S) are considerable (S≈$100 US). In these cases, the advantage of being a member was very substantial. Savings were also considerable for women who delivered at a healthcare centre (S≈$12 US) or who went directly to a hospital maternity unit (S≈$30 US), since deliveries in themselves were already more expensive. For hospitalized patients, the average savings for MHO members was around $35 US at each of the hospitals. Finally, the analyses did not reveal any detectable substitution effect in the expense items.

## Discussion

### Limitations of the Study

To minimize the risks of bias due to self-selection of members and of service users, we controlled for heterogeneity in two ways,: first, by matching members and non-members of MHOs, then by controlling for key covariates in statistical models. Matching variables were selected for each group on the basis of the literature and data availability. Adjustments included modifying factors related to the health facilities and to the users recruited. Moreover, we opted for conservative interpretations based on bilateral testing. Of course, these precautions do not categorically eliminate the possibility of over-attribution of observed results to MHO membership. Still, we believe the risks of distortion from selection bias have been reasonably minimized, an opinion reinforced by the stability of the modelizations and the convergence of the analyses, with and without adjustments. The size of the sample is also a potential limitation of this study. Having more pairs and triads would have increased the power of the analyses. We cannot exclude the possibility that more distinct effects might have been identified. Unfortunately, as it was, even a prospective study involving a year of data collection required considerable effort.

### Major Economic Benefits of Offered by MHOs

The most striking result had to do with the size of the savings achieved by members for an episode of care, even taking into account membership fees. A parturient covered by an MHO paid, on average, one-third less than a non-member parturient. This proportion could reach 40% if she was hospitalized. Comparable patterns have been reported in other studies in Africa [5], [7], [9], [23] and Asia [14]. Hospitalized patients also benefited from MHO membership and experienced substantial savings. Some studies [12], [31] have suggested that MHO membership increases patients’ direct costs during an episode of care, due to the administration of more expensive treatments by health personnel, who know that expenses incurred by the patient will be covered by the MHO. However, the evidence for such a statement in the African context is still too limited. There was nothing in our Benin study to suggest such practices.

### Evidence Still Needed for Other Possible Advantages

The members and non-members who participated in our study were healthcare service users who had already managed to overcome potential barriers to access. Thus, we did not expect to see significant differences on this point. Since they did not have to pull together the funds required to pay for care, members could potentially have consulted sooner. Yet our results did not show this, and few studies have been published on these questions. Was it because the advantages related to financial accessibility were not sufficient to counteract the geographic barriers [17] encountered by those patients who tended to delay seeking services? Or was it due to practices specific to the health-seeking context around childbirth or hospitalization? Two studies of health-seeking behaviours in Uganda, for malaria [32] and episodes of illness in general [33], reported earlier use of healthcare services among MHO members. An in-depth study of health-seeking behaviours might provide more detailed information about the possible impact of MHO membership on the timing of service use.

On average, member parturients were discharged from healthcare centres sooner after delivery than non-members. On the other hand, member parturients sent to a hospital for an obstetrical complication stayed longer in the maternity unit. However, these differences were no longer statistically significant after adjustment through modelling. Our studies do not allow us to draw clear conclusions regarding the impact of mutual membership on length of stay. It may be that parturients are better managed by staff in maternity units, and that members’ families are not pressured to seek rapid discharge, since the qualitative study showed that healthcare workers’ relationships with members were more egalitarian than those with non-members [26]. There as well, the literature was not very useful for putting our results in perspective. Other studies are needed to explore further the impact of insurance coverage on length of treatment in health facilities.

The difference between members’ and non-members’ assessment of care and services received was minimal, primarily because of the respondents’ high (and regularly described elsewhere [34], [35], [36]) levels of satisfaction. However, the members reported a less positive reception at one of the two hospitals. We questioned local authorities, members of the community, and MHO representatives about these observations. This dissatisfaction stemmed from repeated incidents between members and the hospital’s accountant, who was also in charge of billing patients and owned an informal drug dispensary. Members accused him of misappropriating funds in their cases. We ourselves noted a discrepancy between the charges patients reported paying on discharge and the amounts indicated in the payment registries that was significant, recurrent, and limited to this hospital. It is therefore likely that this observed dissatisfaction with how they were received was due to local circumstances rather than to a general propensity to treat MHO members less favourably. In Tanzania, people reported noticing an improvement in healthcare quality in areas covered by community-based insurance [37]. However, in Mauritania, parturients who were MHO members reported dissatisfaction with some of the services received [38]. These results also show the potential limitations of the MHOs’ role in managing the interface between users and healthcare services if the State does not adequately fulfill its role as healthcare system regulator.

### Conclusions

In its 2010 annual report, WHO [1] encouraged risk-sharing in all segments of the population, so as to move toward universal healthcare coverage and to be able to remove user fees. Some have proposed that mutual health organizations could serve as the starting point for progressive construction of national medical insurance systems. Levels of penetration of community-based insurance are still generally low across the continent [39], but the Ghanaian and Rwandan experiences are put forward as encouraging attempts at integrating mutual health organizations in order to achieve universal access to healthcare [1]. These experiences could inspire Benin, whose Head of State officially announced, in December 2011, a universal health insurance system [40]. The results of this study confirm the merits of mutual health organizations for protecting households financially, even if benefits for the poor cannot specifically be determined. As such, health authorities and their international partners would be well advised to consider carefully the possibility of incorporating mutual health organizations into any measures planned to achieve universal insurance. The search for scientific evidence through independent studies should nevertheless continue, in order to understand their impacts more clearly, particularly with regard to services obtained by their members.
